# What really makes residents tick or burn out? Insights from a National survey

**DOI:** 10.1186/s12909-024-06331-z

**Published:** 2024-11-26

**Authors:** Ofira Zloto, Maxim Henenfeld, Orly Weinstein

**Affiliations:** 1 Faculty of Medicine, Tel-Aviv University, Tel-Aviv, Israel; 2 Goldschleger Eye Institute, Sheba Medical Center, Tel-Hashomer, Israel; 3 Clalit Health Services, Tel-Aviv, Israel; 4 Faculty of Health Sciences, Ben-Gurion University of the Negev, Ben-Gurion Ave, Beer Sheva, Israel

**Keywords:** Residency, Residents, Satisfaction rate, Burnout

## Abstract

**Background:**

To examine the satisfaction rates and burnout feelings amongst residents physicians in different specialties and to examine associated affecting factors.

**Method:**

Clalit Health Services (CHS) is the largest health maintenance provider in Israel. A survey was designed by an interdisciplinary team with questions addressing residency and the balance between residency and personal life, as well as the balance between residency and personal life, and was sent to all residents of CHS via personal email from March to May 2022.

**Results:**

Four hundred sixty seven residents completed the survey. The mean satisfaction rate from the residency was 7.4 (± 1.93, 2–10). The highest satisfaction rate from residency was amongst anesthetics, psychiatrists and ophthalmologists ( 8.6, 8.1 and 8.0, respectively) while the lowest was among general surgeons, oncologists, plastic surgeons and orthopedic surgeons (6.8, 6.8, 6.7, 6.1, respectively). There was no correlation between satisfaction rates and monthly working hours or number of overnight shifts. Seventy four percent feel frequent burnout due to high workload and 68% feel frequent burnout due to high number of administrative tasks. Seventy three percent chose the work environment in their department as most influential of their residency experience, 61% chose relations between attending physicians and residents as most significant, and 60% chose the education during the residency as most influential during their residency training.

**Conclusions:**

Satisfaction rates from the residency training require improvement, with differences between the residencies. Number of working hours and number of overnight shifts did not correlate with the satisfaction rates. The factor that influences the most between satisfactory rate and less burnout is the relationship with colleagues and good training programs. Major efforts should be done to improve these factors.

**Supplementary Information:**

The online version contains supplementary material available at 10.1186/s12909-024-06331-z.

## Introduction

The origin of the word “residency” comes from the old times when the physician residents lived in the hospital while undergoing specialist training [1]. Since then, there has been there has been significant changes in the working hours of residents. In Europe, the European Working Time Directive (EWTD) [2] set that the maximum work week of residents is 48 h with a minimum rest period of 11 consecutive hours per 24-h duty and a minimum rest period of 24 h per 7-day duty, or 48 h of rest per 14-day duty [2]. In United Kingdom (UK), the EWTD first applied to consultants due to the concern that the National Health Service (NHS) would not be able to cope with losing so many junior doctor hours, but the EWTD was extended to cover them in August 2004 [3]. In the United States, in September 2010, the Accreditation Council for Graduate Medical Education (ACGME) released new standards of a maximum of 80 duty hours per week, for first-year post-graduate residents, a maximum shift length of 16 h and for intermediate-level residents, a 24-h limit on continuous duty [4]. In Israel, the working hours standards for residents, since 2011, set a maximum of 71.5 duty hours per week with a maximum of 26 h of overnight shifts [5].

Resident’s wellness has gained significant attention in recent years and has become a focus of medical education. There is a worldwide discussion regarding the balance between reducing the working hours of residents and its effect of that on their training, burnout and satisfaction level [6–8]. The ACGME and the American Medical Association (AMA) are developing guidelines and wellness programs to help training programs recognize and mitigate burnout and trainee distress [9]. In Canada, “commitment to self” (physician health and well-being) was recently added as a professionalism sub-competency in the CanMEDS framework [10].

Therefore, there is currently a significant debate in the literature regarding working hours, residency quality, changes in resident generations, their ability to achieve competence in residency, and their feelings of burnout. The purpose of this study was to examine satisfaction rates and feelings of burnout among residents across different specialties, as well as to explore the factors that affect these outcomes.

## Methods

### Study design and participants

Clalit Health Services is the largest health maintenance provider in Israel, providing a broad assortment of healthcare services for a population of roughly 4.7 million inhabitants as of December 2021. CHS is the owner of 14 hospitals (8 general hospitals, 1 pediatric hospital, 3 rehabilitation and geriatric hospitals and 2 psychiatric hospitals) as well as 1,600 community clinics around Israel. In those clinics and hospitals, as of February 2022, 1933 residents are trained in all medical specialties.

This is a survey study (quantitative research strategy). A survey (in Hebrew) was designed and validated by an interdisciplinary team including, the head of Clalit Health Services hospitals, senior doctors, human resources specialists and developmental psychologists (CVR-0.8; S-CVI-0.8; Cronbach's alpha-0.8) (Supp.1). Questions addressed residency training as well as the balance between residency and personal life, including satisfaction from residency, overall satisfaction from residency, frequency and cause for burnout, and factors that most influenced residency experience.

A link to an online version of the survey was sent to all residents of CHS via their email from March to May 2022. Reminders were sent to non-respondents twice to increase response rates. Once the resident completes the survey, they cannot access the link again. All responses were kept confidential.

The study was approved by the Clalit institutional review board (IRB) of the Clalit Health Services. The study was performed in accordance with the ethical standards as laid down in the Declaration of Helsinki and its later amendments or comparable ethical standards. An informed consent was obtained from all subjects.

### Statistical analysis

Quantitative variables were described as mean ± standard deviation. Categorical variables were described as absolute and relative frequencies. Results of questions were compared using t-test analysis evaluating for each of the individual responses. The correlation between satisfaction rates and burn out feelings and different associated factors were tested with Pearson's correlation analysis. Two-tailed *P*-values less than 0.05 were considered statistically significant, whereas results with 95% confidence intervals (CI)s were reported where applicable.

All statistical analyses were performed using SPSS software, version 25 (SPSS, Armonk, NY: IBM Corp).

## Results

### Demographics

Four hundred sixty seven residents (256 men, 211 women) completed the survey. Their mean age was 34.3 years old (± 4.06, 27–52).

Four hundred thirty four residents (93%) worked in a full-time job while 33 residents (7%) worked in a part time job [Table 1 summarizes the characteristics of the cohort].

**Table 1:** Characteristics of the sample

Characteristics	Number	Percentage
**Year of residency**		
Year 1	84	18
Year 2	103	22
Year 3	93	20
Year 4	79	17
Year 5 or more	103	23
**Residency**		
Internal medicine	82	17
Pediatric	63	13
Gynecology and obstetrics	40	9
Anesthesia	35	7
General surgery and subspecialties	35	7
Orthopedic surgery	24	5
Psychiatry	24	5
Ophthalmology	17	4
Geriatrics	15	3
Ear, nose, and throat (ENT)	15	3
Neurology	15	3
Plastic surgery	14	3
Radiology	12	3
Emergency medicine	12	3
Orology	11	2
* Oncology*	8	2
Pathology	7	1
Dermatology	6	1
Rehabilitation	4	1
Not specified	28	6
**Type of hospital**		
Tertiary general hospital	228	49
Primary and secondary general hospital	203	43
Psychiatric hospitals	18	5
Rehabilitation and geriatric hospitals	13	3

### Satisfaction rates’ from residency

**Fig. 1:**
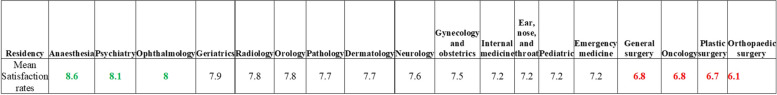
Satisfaction rates per residency

The mean satisfaction rate from the residency was 7.4 (± 1.93, 2–10). The highest satisfaction rate from residency was amongst anesthetics, psychiatrists and ophthalmologists ( 8.6, 8.1 and 8.0, respectively) while the lowest was among general surgeons, oncologists, plastic surgeons and orthopedic surgeons (6.8, 6.8, 6.7, 6.1, respectively) [Fig. 1 summarized satisfaction rates per residency].

The satisfaction rates from residency were significantly lower amongst residents who experienced separation from spouse (6.5 vs 7.5, t-test, *p* < *0.01*), started to take new medications (6.6 vs. 7.6, t-test, *p* = *0.01*) or gain weight (7.1 vs. 7.8, t-test, *p* = *0.01*). The satisfaction rates among residents physicians who considered resigning from their residency were lower compared to residents who did not (5.6 vs. 7.9, t-test, *p* < *0.01*). Moreover, satisfaction rates were lower amongst residents that at the end of the residency do not want to work in the profession of their residency (5.3 vs 7.7, t-test, *p* < *0.01*) as well as those that preferred to work in community clinics compared to those that desired to work in a hospital setting (6 vs. 8, t-test, *p* < *0.01*).

The mean monthly working hours was 176.11 (± 40.40, 13.54–276.58). The mean overnight shifts per month was 3.5 (± 1.96, 0–9.75). There was no correlation between satisfaction rates and monthly working hours (Pearson correlation, *p* = *0.222*). Furthermore, there was no correlation between satisfaction rates and number of overnight shifts ( Pearson correlation*, p* = *0.096*).

### Burnout situations

**Fig. 2:**
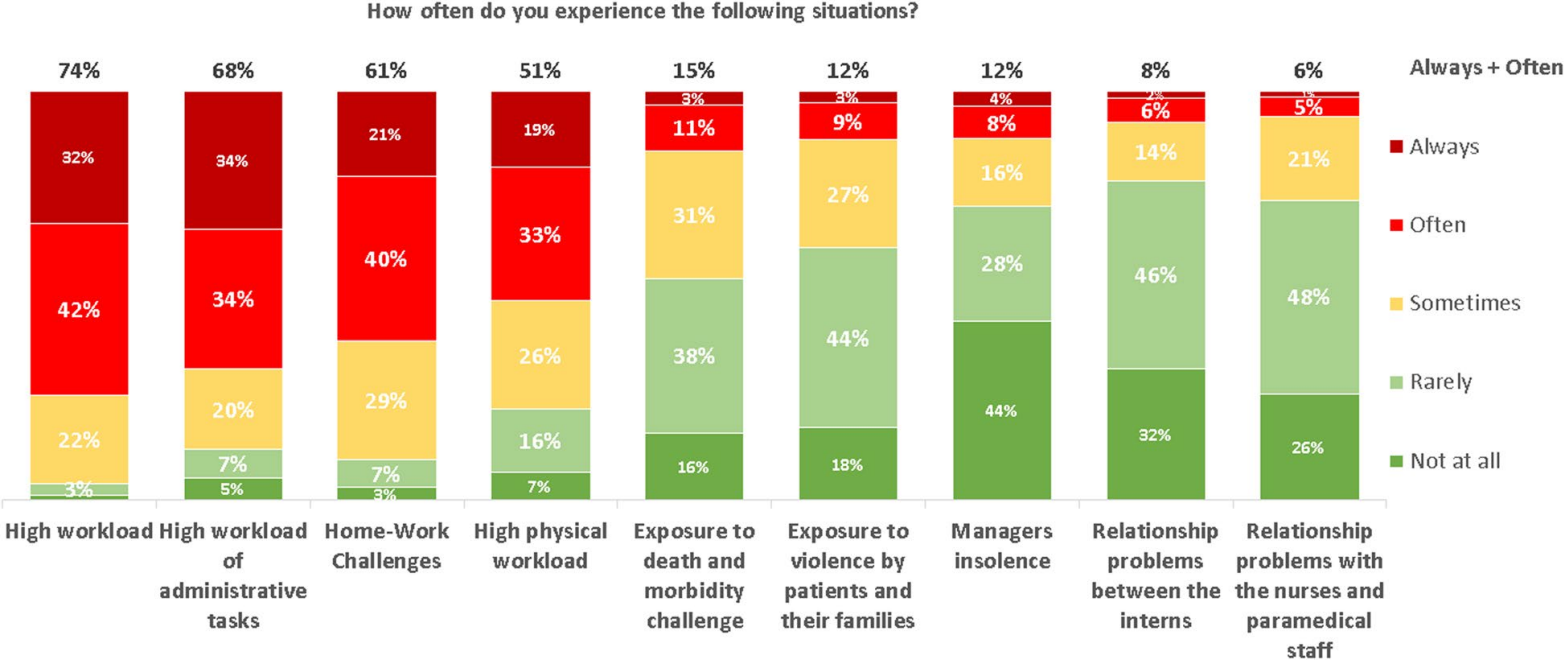
The frequency of burnout situations. Different burnout situations and their frequent

Resident physicians rated the frequency of situations that cause them to feel burnout. Seventy four percent feel frequent burnout due to high workload, 68% feel frequent burnout due to high number of administrative tasks, and 61% feel frequent burnout due to the difficulty to balance between work life and family life. A minority of the residents feel frequent burnout due to communication problems with nurses and para-medical staff (6%), conflicts with co-residents (8%), experienced intimidation and harassment by bosses (12%) and violent behavior of the patients and their families (12%). Figure 2 summarizes the frequency of burnout situations.

There was a significant correlation between high satisfaction rates from residency and low frequency of experiencing intimidation and harassment by bosses, conflicts with co- residents and communication problems with nurses and para-medical staff [Table 2 summarized the correlation between satisfaction rates from residency and frequency of situations that cause residents to feel burnout].

**Table 2:** Correlation between satisfaction rates and frequency of situations that cause burnout

	Intimidation and harassment by bosses	Conflicts with co- residents	Communication problems with nurses and para-medical staff	High administrative tasks	Difficulty to balance between work life and family life	High physical load	High workload	Exposure to violence	Exposure to death
Pearson Correlation	.514^**^	.395^**^	.275^**^	.259^**^	.255^**^	.248^**^	.198^**^	.181^**^	.104^*^
Sig. (2-tailed)	.000	.000	.000	.000	.000	.000	.000	.000	.000

**Statistically significant

*mistake should be *

### The affecting factors of residency

**Fig. 3:**
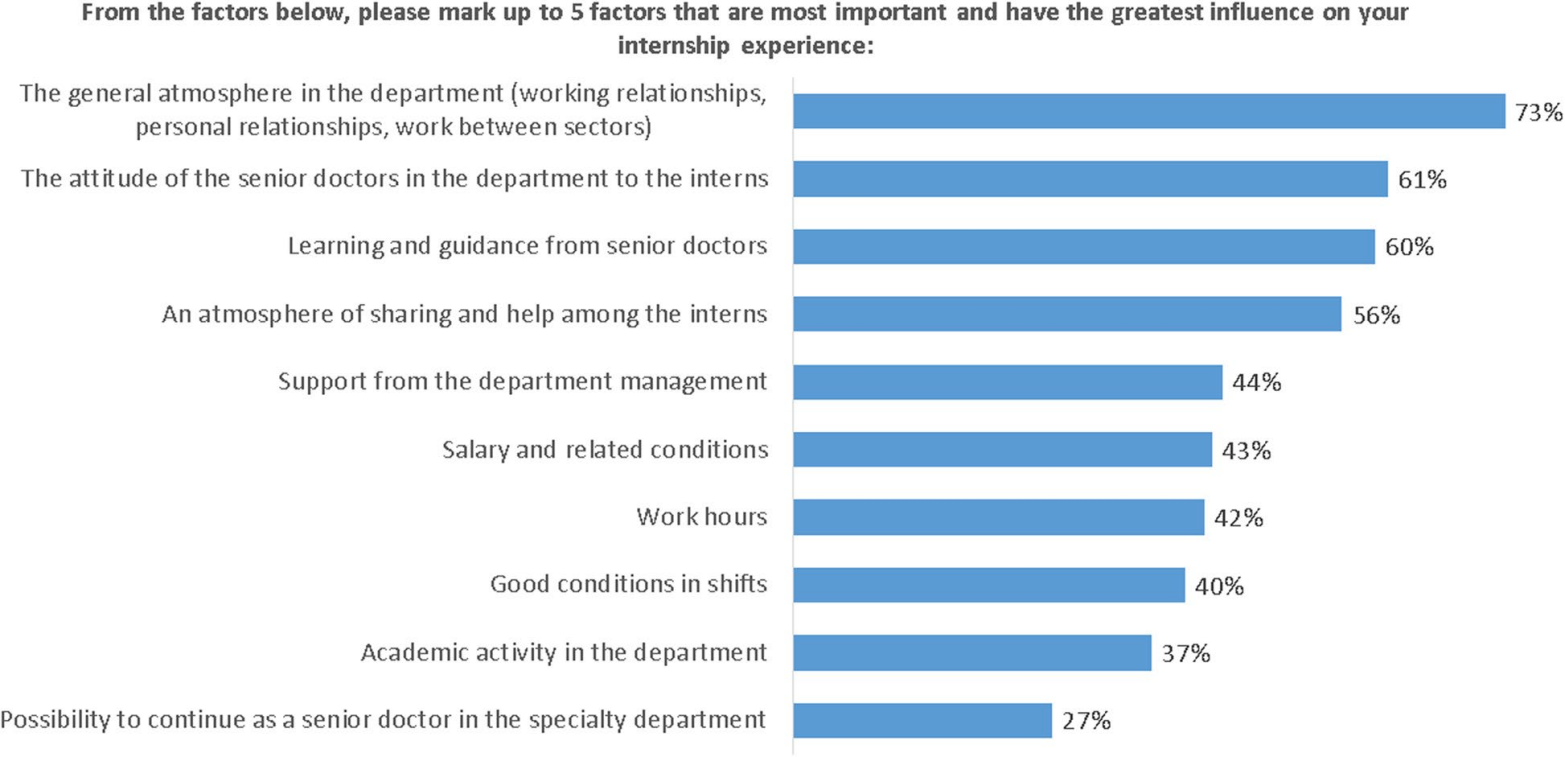
Subjects that influence residency. Residents were asked to select 5 out of 10 factors that most influence their residency experience. The figure illustrates the average score for each factor

The residents were asked to choose 5 out of 10 factors that influence their residency the most. Seventy three percent chose work environment in the department as influenced the most, 61% chose the relations between the senior doctors to the residents and 60% chose education during residency as most influential during their residency training. Only 37% chose the academic work in the department as most influential during residency and only 27% chose the option to be a senior doctor in the department at the end of training as most influencing their residency. Figure 3summarizes factors that influence residency according to the residents' responses..

## Discussion

Residents have a crucial role in the hospital in addition to being the future physician workforce. Burnout in physicians is a threat not just to their personal well-being and patient care but also to the future of the health system in a country. Therefore, in the last years, significant efforts to reduce the feeling of burnout and increase the satisfaction rates from residency have been implimented [6, 9, 10].

In this current study, we found that the mean satisfaction rate from residency was 7.4 out of 10. Most of the residents felt burnout due to the high workload. There was a correlation between high satisfaction rates and low conflicts with colleagues. In accordance, the most influencing factors that affect residency training is the atmosphere in the department.

A mean satisfaction rate from the residency of 7.4 means that most of the residents are overall satisfied with their training programs however that room for improvement exists. Anesthesiologists, psychiatrists and ophthalmologists were the most satisfied residents while orthopedics, general surgeons and plastic surgeons were the least. Those findings are surprising as plastic surgery, general surgery and orthopedics surgery are considered very popular and competitive specialties [11, 12]. One would think that this may rise from the long hours that surgeons work (sometimes more than 10 per day or more than 26 h for overnight shift if they want to stay for a spacific surgery). However, we did not find any correlation between satisfaction rates to monthly working hours and numbers of overnight shifts. Furthermore, we found a correlation between low satisfaction rates and residents medical problems as well as personal relationship difficulties, which been reported in the past [13, 14].

According to the latest employment agreements in Israel, a resident is expected to perform an average of 6 overnight shifts per month and work 45 h per week. In this study, the mean monthly working hourswas 176.11 (44 h per week) and 3.5 overnight shifts per month. In recent years, the reduction of working hours for residents in Israel has sparked significant debate. Some argue that long working hours are a major source of stress for resident physicians, while others contend that reducing working hours hinders residents' ability to achieve competency in their medical specialty [15, 16]. In this study, we found no correlation between working hours and satisfaction rates, as well as feelings of burnout. This result suggests that residents' perceptions of how they spend their working hours may be more significant than the total number of hours worked. findings together with other studies in the past literature [15, 16] may suggest that simply reducing work hours may be insufficient to reduce burnout, and it may be essential to focus on the types of stress felt by each resident and specialties.

We found that the burnout feeling is mainly due to high workload and poor work–family life balance. Due to the shortage in doctors as well as shortage in health teams in Israel, the clinical and bureaucratic workload on the residents is pretty high [17]. Furthermore, responsibilities outside of the hospital including active participation in research, answering emails, or studying further consumes limited time resident physicians may have.

Residents in Israel usually start their residency when they are in their early or late 30th, so during the residency they usually have a spouse and small children. Therefore, continuing to work at home, probably increases their fatigue feeling as well as impaired their work–family balance. Efforts should be done to decrease workload in hospitals and to implement policies to improve work– family life balance like no answering emails on weekends, protected hours for research and studying, etc.

Some burned out situations cause the residents to feel more burn out like conflicts with colleagues and harassment by bosses. Low frequency of conflicts with medial staff (residents, nurses and para-medical staff) as well as low frequency of experiencing intimidation and harassment by bosses was found to be correlated with higher satisfaction rates. Moreover, when the residents, in this survey, needed to choose 5 most important factors that influence their residency, the three most chosen factors were the relationships with colleagues and the training. This is in accordance to previous studies. Saijo et al. found that support from co-workers had significant protective odds ratios for depressive symptoms. Moreover, Matsuo et al. found that communication problems at the workplace was independently associated with burnout and stress [13, 18]. Therefore, health system managers should focus mainly on improving working- relationship in departments, improving residency programs and reducing abuse and harassment. Formal and informal curriculum and workshops for all members of the healthcare system should be performed in order to improve this.

Limitations of this study include that the given survey was sent after two years following the COVID-19 pandemic, which significantly impacted the health system, the work life and family life were different from regular times. Therefore, we send this survey one year following the last lockdown in Israel. In addition, as with any survey, our study struggles with response bias, or example, compliance may have been affected by burnt-out residents apathetic to filling out surveys, or alternatively are overly inclined to fill out the survey. However, still a high response level was achieved from different specialties.

## Conclusions

We found that satisfaction rates among residents indicate a need for improvement, with variations observed between different residencies. The number of working hours and overnight shifts did not correlate with satisfaction rates. The factors that most significantly impact satisfaction and reduce burnout are relationships with colleagues and the quality of training programs. Further studies should be conducted globally to verify the results of this study. Significant efforts should be made to enhance these factors, as they contribute to burnout and feelings of dissatisfaction among residents.

## Supplementary Information

Supplementary Material 1.

## Data Availability

No datasets were generated or analysed during the current study.

## References

[1] Tolkien JRR. Oxford english dictionary. 1973.

[2] Department of Health UK D of HU. What is the European working time directive? http://webarchive.nationalarchives.gov.uk/+/www.dh.gov.uk/en/Managingyourorganisation/Workforce/Workforceplanninganddevelopment/Europeanworkingtimedirective/DH_077304.

[3] JG T. Time for training: a review of the impact of the European working time directive on the quality of training. London: Department of Health; 2010.

[4] Accreditation council for graduate medical education. Common program requirements. The ACGME. 2024. https://www.acgme.org/programs-and-institutions/programs/common-program-requirements/.

[5] Ministry of Justice R. Notification of an amendment to the general permit for work during weekly rest periods and overtime in medical facilities and institutions for the care of the elderly or children (temporary provision). COMMISSION STAFF WORKING PAPER.

[6] Quirk R, Rodin H, Linzer M. Targeting causes of burnout in residency: an innovative approach used at Hennepin healthcare. Acad Med. 2021;96(5):690–4. 10.1097/ACM.0000000000003940. 33496434 10.1097/ACM.0000000000003940

[7] Nishimura Y, Miyoshi T, Obika M, Ogawa H, Kataoka H, Otsuka F. Factors related to burnout in resident physicians in Japan. Int J Med Educ. 2019;10:129–35. 10.5116/ijme.5caf.53ad. 31272084 10.5116/ijme.5caf.53ad PMC6766397

[8] Ferguson C, Low G, Shiau G. Resident physician burnout: insights from a Canadian multispecialty survey. Postgrad Med J. 2020;96(1136):331–8. 10.1136/postgradmedj-2019-137314. 32123129 10.1136/postgradmedj-2019-137314

[9] Education. AC for GM. Improving physician well-being, restoring meaning in medicine. https://psnet.ahrq.gov/issue/physician-well-being.

[10] Frank JR, Snell LS SJ. Draft CanMEDS 2015: physician competency framework—Series II. Canada R Coll Physicians Surg Canada. Royal College of Physicians and Surgeons of Canada.

[11] AAMC. AAMC Careers in Medicine. https://careersinmedicine.aamc.org/.

[12] Fazel S, Ebmeier KP. Specialty choice in UK junior doctors: is psychiatry the least popular specialty for UK and international medical graduates? BMC Med Educ. 2009;9(1):77. 10.1186/1472-6920-9-77. 20034389 10.1186/1472-6920-9-77 PMC2805648

[13] Matsuo T, Takahashi O, Kitaoka K, Arioka H, Kobayashi D. Resident burnout and work environment. Intern Med. 2021;60(9):1369–76. 10.2169/internalmedicine.5872-20. 33281158 10.2169/internalmedicine.5872-20 PMC8170257

[14] Zumbrunn B, Stalder O, Limacher A, et al. The well-being of Swiss general internal medicine residents. Swiss Med Wkly. 2020;150: w20255. 10.4414/smw.2020.20255. 32557425 10.4414/smw.2020.20255

[15] YNET. The struggle of the interns. https://www.ynet.co.il/topics/מאבק_המתמחים16.Shumfalvi. Accessed 2 Aug 2022.

[16] Shumfalvi A. Representative of the interns: "We will have to enter another round of struggle." Ynet. Published online April 14, 2022. https://www.ynet.co.il/health/article/rjcqdvhnc. Accessed 2 Aug 2022.

[17] Health at a Glance 2021. OECD; 2021. 10.1787/ae3016b9-en.

[18] Saijo Y, Chiba S, Yoshioka E, et al. Effects of work burden, job strain and support on depressive symptoms and burnout among Japanese physicians. Int J Occup Med Environ Health. 2014;27(6):980–92. 10.2478/s13382-014-0324-2. 25503892 10.2478/s13382-014-0324-2

