# Supplementary material for: What really makes residents tick or burn out? Insights from a National survey
Source: BMC Med Educ. 2024 Nov 26;24:1377. doi: 10.1186/s12909-024-06331-z (PMC11600643; doi:10.1186/s12909-024-06331-z)
Supplement: Supplementary file 1 — Supplementary Material 1. [file 12909_2024_6331_MOESM1_ESM.docx]

**Hello,**

A short questionnaire about your residency, the degree of support you are given in and outside of the residency, emotional difficulties during the residency and quality of life is attached.

The questionnaire asks for your personal position or thoughts, there are no right or wrong answers here. Your participation in the study is anonymous and on a voluntary basis. You can skip questions if they cause you discomfort.

This study will also be conducted in other hospitals of the Clalit Health service and its purpose is to learn about the coping and difficulties of the residents during the residency. Your participation in the study will not benefit you personally. However, your answers will provide a lot of knowledge about how the interns cope and will help in making various decisions.

Duration of filling out the questionnaire: 5-10 minutes.

**Background questions**

Gender:

1. Male

2. Female

3. Other

Age:

Marital Status:

1. Single

2. Married

3. Divorced

4. A widower

5. In a relationship

6. Other

Do you have children under the age of 18?

Yes

No

**Specialization:**

Specialization profession:

1. Oncology

2. Public health

3. Geriatrics

4. Anesthesia

5. Newborn and gynecology

6. Urological surgery

7. Orthopedic surgery

8. General surgery

9. Plastic and aesthetic surgery

10. Thoracic surgery

11. Pediatric surgery

12. Vascular surgery

13. Otolaryngology diseases and head and neck surgery

14. Skin and sexual diseases

15. Eye diseases

16. Medical director

17. Neurosurgery

18. Neurology

19. Psychiatry or child and adolescent psychiatry

20. Pathology

21. Radiology

22. Nuclear medicine

23. Emergency medicine

24. Nuclear medicine

25. Internal medicine and subspecialties of internal medicine

26. Physical medicine and rehabilitation

27. Pediatrics

28. Other

A year in internship:

1. First year

2. Second year

3. Third year

4. Fourth year

5. Fifth year

6. Sixth year

7. Seventh year

8. Eighth year

Please rate how often you feel the following situations:

| **Not at all** | **Rarely** | **Sometimes** | **Often** | **Always** |  |
| --- | --- | --- | --- | --- | --- |
|  |  |  |  |  | **Difficulty combining home and work** |
|  |  |  |  |  | **Heavy workload** |
|  |  |  |  |  | **A lot of physical strain** |
|  |  |  |  |  | **Load of administrative tasks** |
|  |  |  |  |  | **Difficulty due to exposure to death and morbidity** |
|  |  |  |  |  | **Exposure to violence by patients and their families** |
|  |  |  |  |  | **Insolence on the part of superiors** |
|  |  |  |  |  | **Relationship problems between the interns** |
|  |  |  |  |  | **Relationship problems with nurses and paramedical staff** |

Please rate the following factors from most important (6) to least important (1) during the internship:

1. Working hours

2. Fair wages and economic conditions

3. Possibility to continue as a senior doctor in the department where I specialize

4. Academic activity in the department

5. An atmosphere of sharing and mutual help between the interns

6. Support from the department management

7. Escort and guidance from senior doctors

8. Favorable conditions in shifts

9. The attitude of the senior doctors in the department to the interns

10. The general atmosphere in the department (working relationships, personal relationships, work between sectors)

Are you thinking of leaving the internship –

Yes

No

At the end of the internship, would you like to continue working in the profession?

Yes

No

Still undecided

At the end of the internship, would you like to continue working in the:

Hospital

Community

Hospital + community

Do not know yet

**Lifestyles**

Did you start taking new medications for a chronic disease during the internship?

Yes

No

Do you smoke?

Yes

No

Do you exercise?

Yes

No

Did you gain weight during the internship?

Yes

No

Did you experience separation from your spouse during the internship?

Yes

No

**Thank you!**
